# Paediatric minor head injury applied to Paediatric Emergency Care Applied Research Network CT recommendations: An audit

**DOI:** 10.4102/sajr.v26i1.2289

**Published:** 2022-04-14

**Authors:** Jacques du Plessis, Sharadini K. Gounden, Carolyn Lewis

**Affiliations:** 1 Department of Diagnostic Radiology, School of Clinical Medicine, University of the Witwatersrand, Johannesburg, South Africa; 2 Department of Emergency Medicine, School of Clinical Medicine, University of the Witwatersrand, Johannesburg, South Africa

**Keywords:** paediatric trauma, computed tomography (CT), minor head injuries, PECARN, traumatic brain injury, diagnostic reference levels, low- and middle-income countries, ionising radiation

## Abstract

**Background:**

Traumatic brain injury (TBI) is a common cause of paediatric morbidity and mortality, with higher TBI rates in low- and middle-income countries. Non-contrast brain CT is the gold standard for diagnosing intracranial injuries; however, it exposes patients to ionising radiation. The Paediatric Emergency Care Applied Research Network (PECARN) clinical decision rule (CDR) aids clinicians in their decision-making processes whilst deciding whether a patient at very low risk of a clinically important TBI (ciTBI) requires a CT scan.

**Objectives:**

To establish whether the introduction of the PECARN CDR would affect CT utilisation rates for paediatric patients presenting with minor blunt head injuries to an academic hospital in Gauteng, South Africa.

**Method:**

This was an audit of paediatric patients who presented with minor blunt head injuries and were referred for CT imaging at an academic hospital in Gauteng, compared with PECARN CDR recommendations, over a 1-year period.

**Results:**

A total of 100 patients were referred for CT imaging. Twenty patients were classified as very low risk, none of whom had any CT findings of a TBI or ciTBI (*p* < 0.01). A total of 61 patients were classified as intermediate risk and 19 as high risk. In all, 23% of the intermediate and 47% of the high-risk patients had CT features of a TBI, whilst 8% and 37% had a ciTBI, respectively.

**Conclusion:**

Computed tomography brain imaging may be omitted in patients classified as very low risk without missing a clinically important TBI. Implementing the PECARN CDR in appropriate patients would reduce CT utilisation rates.

## Introduction

Traumatic brain injury (TBI) is a common cause of paediatric morbidity and mortality with an annual global reported incidence ranging between 47 and 280 per 100 000 children.^1^ Ninety percent of all paediatric TBIs are classified as minor,^2^ which is defined as a Glasgow Coma Scale (GCS) of 14–15.^3^

Non-contrasted head CT is the investigation of choice to diagnose an intracranial injury. In the United States, approximately half of all paediatric patients presenting with a head injury to an emergency department (ED) will be subjected to a CT examination.^4^ Computed tomography scans are the largest contributor to diagnostic radiation, with usage doubling between 1995 and 2005 in paediatric patients in the United States.^5^ The detection of life-threatening diagnoses, however, has not changed despite the increased utilisation of CT examinations. The incidence of positive CT findings is less than 10% of examinations performed on patients with minor TBI.^6,7^

Population-based studies have illustrated higher TBI rates in low- and middle-income countries compared with high-income countries.^8^ Despite an estimated 8 million TBI cases per year in Africa, most of which occur in patients who are less than 40 years of age (incidence 801 per 100 000 persons), there is a paucity of African paediatric-specific TBI statistics.^9^

Paediatric patients with TBI create a diagnostic dilemma for clinicians since they present with different signs and symptoms in comparison with adults due to age-related physiological and anatomical differences. It can thus be challenging for clinicians to confidently assess the paediatric patient’s neurological status and evaluate for signs of a TBI.^4,6,10^

Treating clinicians need to balance the relative risks and benefits when deciding whether a patient requires a CT examination after sustaining minor blunt head trauma.^11^ The rapid diagnosis of an intracranial injury is vital to patient management;^6^ however, CT examinations expose patients to ionising radiation, which may result in deoxyribonucleic acid (DNA) damage.^12^ Paediatric patients are more susceptible to the effects of ionising radiation because of their rapid cellular turnover rates and longer life expectancy, which results in an increased risk of radiation-induced cancers when compared with adults undergoing a similar examination.^11^

Diagnostic reference levels (DRLs) were introduced by the International Commission on Radiological Protection (ICRP) in 1996 to monitor procedure-specific radiation doses and thereby set the standard for acceptable clinical practice.^13^ Dose length product (DLP) and volume-based CT dose index (CTDI_vol_) are two indicators used to evaluate DRLs and quantify patient exposure to ionising radiation.^11,12^ The CTDI_vol_ defines the mean dose per image slice, whilst the DLP is the product of the total scan length and CTDI_vol,_ representing the total energy absorbed along the length of the scan.^13^ Many high-income countries have fixed regulations regarding the establishment and maintenance of DRLs; however, in low- and middle-income countries, a similar practice has not been widely adopted, with equipment and maintenance constraints cited as the most common reasons.^13^ A retrospective audit by Van der Merwe et al. reviewed paediatric DRLs of different CT examinations performed at two South African University Hospitals and compared their findings with international DRLs to establish local DRL values for the University of the Witwatersrand academic hospitals and their referral hospitals. They defined local DRL values as the 75th percentile of data distribution for different CT examinations. The study concluded that most of the DRLs were acceptable and internationally comparable suggesting that effective protocols and techniques are in place. However, they did observe an increase in DLP values during after-hour studies at both hospitals, thought to be attributed to reduced staff members, particularly senior trained staff, experienced in imaging paediatric patients.^14^

Multiple clinical decision rules (CDRs) are available to aid clinicians in their decision-making process when managing patients with a suspected TBI.^15^ The Paediatric Emergency Care Applied Research Network (PECARN) CDR is applied to paediatric patients presenting with minor blunt TBI, aiming to identify patients in whom there is no clinically important traumatic brain injury (ciTBI) and who do not require a CT brain scan.^15^ Clinically important traumatic brain injury is defined as an injury resulting in patient demise, intubation and ventilation for a period greater than or equal to one day; need for neurosurgical intervention; or hospital admission for observation for a period greater than or equal to two days associated with a TBI on CT.^4^

**FIGURE 1:**
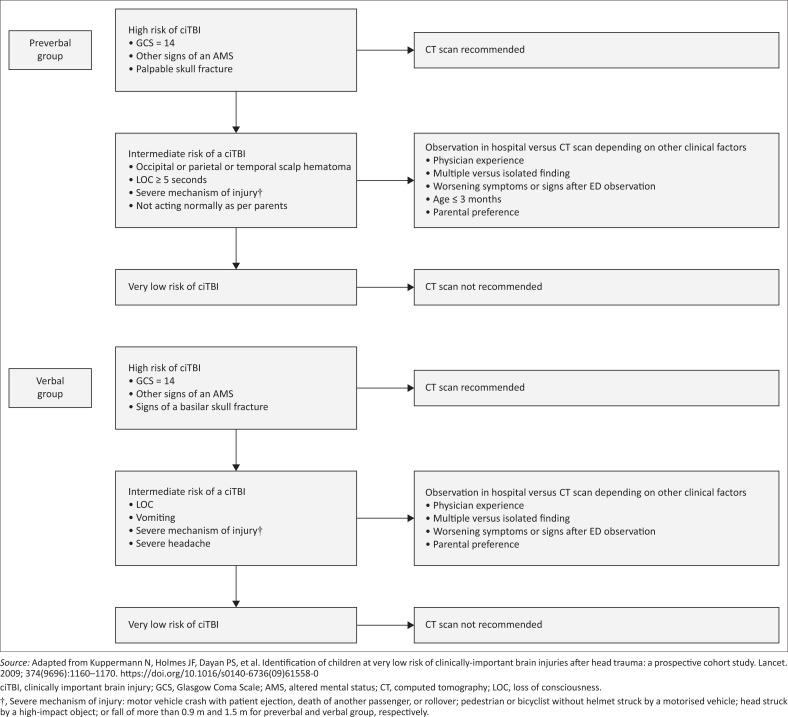
Paediatric Emergency Care Applied Research Network clinical decision rule for preverbal and verbal group.

The PECARN CDR was derived from a large, prospective cohort, which made it possible to develop two separate CDRs, one for preverbal patients (less than 2 years of age) and one for verbal patients (aged two years or more).^4,15^ Its use has been internally and externally validated, including in low- and middle-income countries.^16^ Nakhjavan-Shahraki et al. evaluated the PECARN CDR in Iran and concluded that it had a sensitivity of 92.3% and 100.0% in predicting ciTBI in the preverbal and verbal groups, respectively.^16^ The PECARN CDR risk stratifies patients into one of the three groups: high, intermediate and very low risk and advises on whether CT imaging is required (see Figure 1).^4,17^

In the United States, approximately 50% of all paediatric patients presenting with head trauma, irrespective of the severity of their injury, will be subjected to a CT scan. Lower CT rates are observed in EDs using a CDR or who have active quality improvement programmes.^4,11^ There is a paucity of data for the South African setting; however, given the international trend, implementing a CDR to aid clinicians to confidently exclude ciTBI would potentially reduce CT scan utilisation rates in paediatric patients presenting with minor TBI.

The aim of this study was to establish whether the introduction of the PECARN CDR would affect CT utilisation rates for paediatric patients presenting with minor blunt head injuries to a university affiliated academic hospital in Gauteng, South Africa. Further objectives were to quantify the extent of potentially unnecessary CT imaging, as determined by both a normal CT scan and an absence of a ciTBI and assess whether the patient’s exposure to ionising radiation is within suggested local DRLs.

## Methods

This retrospective, descriptive record retrieval study analysed data from the paediatric emergency unit (PEU) at an academic hospital in Gauteng, an urban quaternary health facility that provides 24-h services to patients from birth to 16 years of age and attends to approximately 12 000 patients annually.

Patients aged 16 years or less, who presented to the PEU within a 24-h period after sustaining a minor blunt head injury, which is defined as an initial GCS of 14–15 and referred for CT brain examination over a 1-year period (01 January 2019 – 31 December 2019), were included in the study. Patients who presented with penetrating head injury; who had pre-existing conditions such as brain tumours, neurological disorders, ventricular shunts or bleeding disorders; with suspected non-accidental injury; who received imaging at an outside healthcare facility before transfer or who had insufficient information in their medical records to risk stratify according to the PECARN CDR, were excluded from the study.

Potential eligible patients were identified by reviewing the triage diagnosis recorded in the patient register in the PEU at the academic hospital. Patient files that had a triage diagnosis related to trauma and possible head injury were retrieved in the medical records to determine patient eligibility as dictated by the inclusion and exclusion criteria. Patients were stratified into one of the three PECARN risk groups based on their age and clinical parameters as set out by PECARN CDR (Figure 1) and evaluated to determine whether the patient was referred for a CT examination and whether a ciTBI was present.

Unenhanced CT brain studies evaluated in this study were acquired using vendor-specific paediatric protocols from CT machines currently in use at the academic hospital (Phillips Ingenuity 128 slice, Philips Brilliance 64 slice and Siemens Somatom 64 slice).

The CT brain examinations for eligible patients were re-read on the Picture Archiving and Communication System (PACS), by two radiologists and one radiology registrar, to establish whether there were any CT signs of TBI as defined by the PECARN CDR. The findings of these readings were concordant amongst the readers. Positive CT findings of a TBI were defined by the presence an intracranial haemorrhage or contusions, cerebral oedema, traumatic infarction, diffuse axonal injury, shearing injury, sigmoid sinus thrombosis, midline shift of intracranial contents or herniation, skull diastasis, pneumocephalus or a skull fracture depressed by at least one width of the skull table. The DLP and CTDI_vol_ for each CT examination were also evaluated.

The data were analysed using IBM^®^ statistical package for the social sciences (SPSS) Statistics version 25. Descriptive statistics are presented as frequencies and percentages for categorical variables. For continuous variables, means and standard deviations summarise normally distributed data whilst medians and ranges summarise non-normally distributed data. Associations between categorical variables were examined using the Pearson chi-square test. The Mann–Whitney U test and the Kruskal–Wallis test were used to compare results from continuous scale variables. Significance testing was set at the 95% confidence.

### Ethical considerations

Ethical clearance to conduct this study was obtained from the Human Research Ethics Committee (Medical), University of the Witwatersrand (number: M200356).

## Results

During the study period, a total of 100 paediatric patients with minor blunt TBI were referred for CT imaging at the academic hospital. A total of 65% of patients were male and 35% female. Patients’ age varied between 1 month and 15 years (mean age of 69.4 months). In all, 17 patients (17%) were classified into the preverbal category (mean age of 10.6 months) and 83 patients (83%) into the verbal category (mean age 81.5 months).

Of the 100 patients, 20% were classified as very low risk, 61% as intermediate risk and 19% as high risk for a TBI according to PECARN guidelines. A total of 23% (*n* = 23) patients had a positive CT finding of a TBI as shown in Table 1. There was a statistically significant association between the PECARN score, for both preverbal and verbal groups, and CT features of a TBI, as illustrated in Table 2.

**TABLE 1:** Computed tomography features of a traumatic brain injury parameters present in patients.

CT features of TBI	Number of patients (*n* = 23)	Percentage (%)
ICH or cerebral contusion	18	78
Cerebral oedema	2	9
Traumatic infarction	0	0
Diffuse axonal injury	0	0
Shearing injury	0	0
Sigmoid sinus thrombosis	0	0
Midline shift or brain herniation	2	9
Skull diastasis	3	13
Pneumocephalus	6	26
Depressed skull fracture with depression greater than the width of the table of the skull	10	43

CT, computed tomography; TBI, traumatic brain injury; ICH, intracranial haemorrhage.

**TABLE 2:** Association between PECARN risk category and computed tomography features of a traumatic brain injury.

PECARN risk category	*N*	CT features of TBI				*p*
		Yes		No		
		*n*	%	*n*	%	
Low	20	0	0	100	100	< 0.01
Intermediate	61	14	23	7	77	
High	19	9	47	10	53	

PECARN, Paediatric Emergency Care Applied Research Network; N, number of patients; CT, computed tomography, TBI, traumatic brain injury.

In all, 12% of patients (*n* = 12) had a ciTBI and were admitted for 2 or more days, with 7% (*n* = 7) of patients classified as high risk and 5% (*n* = 5) as intermediate risk. No patients were classified as very low risk had a ciTBI. No patients demised, required intubation and ventilation or neurosurgical intervention. Table 3 demonstrates the statistically significant association between the PECARN score, for both preverbal and verbal groups, and ciTBI.

**TABLE 3:** Association between PECARN risk category and a clinically important traumatic brain injury.

PECARN risk category	*N*	ciTBI				*p*
		Yes		No		
		*n*	%	*n*	%	
Low	20	0	0	100	100	< 0.01
Intermediate	61	5	8	56	92	
High	19	7	37	12	63	

PECARN, Paediatric Emergency Care Applied Research Network; N, number of patients; ciTBI, clinically important traumatic brain injury.

Table 4 demonstrates the 75th percentile of data distribution for DLP and CTDI_vol_ irrespective of the patients assigned PECARN score. Patients were divided into age groups (< 1 year, 1–5 years, 5–10 years and 10–15 years) and DRL values rounded to the nearest single digit for CTDI_vol_ and nearest 5 for DLP, similar to Van der Merwe et al. to compare the results with suggested local DRL values.^14^ None of the patients in our study were aged 16 years, and thus this age group was not included in the age categories.

**TABLE 4:** The 75th percentile dose length product (mGy*cm) and volume-based computed tomography dose index (mGy) values for various age groups.

Diagnostic reference level parameters	Age groups	75th Percentile	95% CI	
			Lower	Upper
DLP (mGy*cm)	0–1 years	360	290.8	755.9
	1–5 years	385	332.8	606.2
	5–10 years	735	508.7	859.5
	10–15 years	945	761.2	1060.3
CTDI_vol_ (mGy)	0–1 years	16	14.7	38.8
	1–5 years	17	15.8	34.6
	5–10 years	35	23.6	38.8
	10–15 years	39	38.5	41.8

CI, confidence interval; DLP, dose length product; CTDI_vol_, volume-based computed tomography dose index.

## Discussion

Paediatric patients with minor blunt head injury commonly present to EDs worldwide, with an upward trend reported over the past decade in the United States.^4,15^ A recent audit by the Pietermaritzburg Metropolitan Trauma Service over a 4-year period included 563 patients, aged 18 years or less, who were treated for a TBI. A total of 96.0% of cases were related to blunt trauma with 80.0% sustaining a minor TBI. The most common mechanisms of injury were pedestrian vehicle accidents (33.0%), interpersonal violence (19.0%) and falls (18.0%). A total of 62 patients (10.6%) required neurosurgical intervention.^18^

In all, 51% of patients in the current study presented with a severe mechanism of injury, with 63% of patients involved in pedestrian vehicle or bicycle accidents. Compared with high-income countries, pedestrian vehicle accidents account for the largest portion of road traffic-related morbidities and mortalities in low- and middle-income countries.^19^

Emergency physicians are tasked with assessing patients with potential TBI and the need to decide whether a patient can be safely discharged or requires further investigation with CT brain imaging to exclude intracranial pathology. A retrospective audit performed at George Hospital in the Western Cape, South Africa, reviewed the appropriateness of CT and MRI requests. The requests were classified, according to the American College of Radiology Appropriateness Criteria (ACR AC), into one of three categories: ‘usually appropriate’, ‘might be appropriate’ and ‘not appropriate’. The ACR AC are evidence-based guidelines intended to assist clinicians on the appropriate use of imaging studies for specific clinical scenarios. Of the 515 CT and MRI studies reviewed, 11.2% were classified as ‘not appropriate’, with CT brain studies being the most inappropriately requested study.^20^

Clinical decision rules have been proven to assist clinicians in their decision-making process.^15^ Clinical decision rules are derived from original research and require at least three variables obtained by reviewing the patients’ history, physical examination and investigations.^15^ Common CDR used in clinical practice include the Canadian Assessment of Tomography for Childhood Injury (CATCH), Children Head Injury Algorithm for the Prediction of Important Clinical Events (CHALICE) and PECARN.^3^

The CHALICE and CATCH CDRs aim to identify patients for whom a CT examination is indicated.^15^ The PECARN CDR is derived from a large and diverse study population that presented with minor blunt head injuries.^4,15^ It is regarded as a suitable CDR for identifying patients at very low risk of ciTBI after sustaining a minor blunt head injury, with a reported sensitivity of 98.6% and 96.7% in preverbal and verbal patients, respectively.^15^ It has a very high negative predictive value (NPV) for identifying patients in whom there is no ciTBI and where workup with CT is not necessary. The NPV with a confidence interval (CI) of 95.0% is 99.9% in the preverbal and 99.9% in the verbal derivation group.^4^

Of the 20 patients (20%) classified as very low risk in our study according to the PECARN score and referred for CT imaging, none had a positive CT finding of a TBI or a ciTBI (*p* < 0.01). Had the PECARN CDR been applied, these imaging studies could have been safely omitted without missing any CT findings of TBI or a ciTBI.

Most patients (*n* = 61; 61%) referred for imaging were classified into the intermediate-risk category. Of these patients, 14 (23%) had a positive CT finding of a TBI, whilst 5 (8%) of the patients had a ciTBI. This is higher than that predicted by the PECARN CDR (risk of a ciTBI predicted to be 0.9% for preverbal and 0.8% for the verbal group).^4^ The association between high-risk classified patients and ciTBI was 37%, which is also higher than that predicted by the PECARN CDR (risk of ciTBI predicted as 4.4% for preverbal and 4.3% for verbal group).^4^

This study retrospectively evaluated and risk stratified patients referred for CT imaging based on their medical records. We did not include all patients who presented to the PEU with minor blunt traumatic head injuries. Patients were assessed and referred for imaging by doctors with varying levels of training and experience. The aforementioned variables and a small sample size (*n* = 100) might account for the discrepancy in the expected and the actual association between intermediate and high-risk groups and CT findings of a TBI and ciTBI.

For patients in the intermediate-risk group, the PECARN rules are assistive, rather than directive.^4^ Shared decision-making, which takes into account clinician, patient and parental preferences, should be implemented when deciding whether a CT scan is required or not.^11^ Specific factors that need to be considered in low- and middle-income countries include access and availability of CT facilities; ED resources such as trained staff, equipment and a designated area for observation; the risk of ED overcrowding and exposing patients and parents to hospital-acquired infections; parental financial pressures and access to safe transport should a patient that was discharged from ED require urgent re-evaluation.

A large percentage of patients in the intermediate group do not require a CT scan if they can be observed in the ED for 4–6 h post-injury. The reported risk of a ciTBI in a patient with an isolated intermediate-risk predictor is low and ranges between 0.2% and 1.4%, depending on the specific PECARN predictor.^11^ In a prospective, multicentre observational study performed by Nigrovic et al., it was concluded that CT utilisation rates were lower in patients with minor TBI who underwent a period of observation than those who were not observed (31.1% vs 35.0%, respectively), with both groups having similar rates of ciTBI (0.75% vs 0.87%).^21^ Our study did not evaluate the clinical factors referring clinicians need to take into account when deciding whether to observe or perform a CT scan in the intermediate-risk group, such as parental preference; physician experience; isolated versus multiple findings and worsening of symptoms or signs after ED observation, as suggested by the PECARN CDR (Figure 1).

Computed tomography scans expose patients to ionising radiation, which is associated with both stochastic and deterministic effects, with the former being the main concern in paediatric patients. Stochastic effects have no threshold radiation dose and include carcinogenesis and genetic mutations.^12^ Paediatric patients are more susceptible to the effects of ionising radiation because of their rapid cellular turnover rates and longer life expectancy, which results in an increased risk of radiation-induced cancers compared with adults undergoing a similar examination.^11^ The risk of carcinogenesis increases with decreasing patient age.^4^ A retrospective study performed in Great Britain followed up 178 604 paediatric patients who had at least one CT study of any type before the age of 22 years. The study concluded that patients who were exposed to ionising radiation at a young age had an elevated risk for developing malignancies after a 10-year follow-up period, specifically leukaemia and brain tumours.^11^

Computed tomography scans should be indicated and justifiable with justification defined by the ICRP as ‘any decision that alters the radiation exposure situation should do more good than harm’.^22,23^ Radiologists should always adhere to the ‘as low as reasonable achievable’ (ALARA) principle to reduce radiation exposure whilst ensuring diagnostic reliability.^12,24^

We compared our 75th percentile DRL values with local DRL values suggested by Van der Merwe et al. as shown in Table 5.^14^ The CTDI_vol_ values, except for 0–1 year and 1–5 year age groups, and DLP values for all age groups were higher than locally suggested DRLs, with the largest discrepancy observed in the 5–10 year and 10–15 year age groups. Thus the majority of patients in this audit were exposed to unnecessary high levels of ionising radiation, which could have long-term health implications.

**TABLE 5:** Comparison of 75th percentile dose length product (mGy*cm) and volume-based computed tomography dose index (mGy) values to suggested local diagnostic reference levels.

Age	CTDI_vol_ 75th percentile (mGy)		DLP 75th percentile (mGy*cm)	
	DP	VDM	DP	VDM
0–1 years	16	21	360	315
1–5 years	17	21	385	365
5–10 years	35	23	735	460
10–15 years	39	33	945	750

*Source:* Adapted from Van der Merwe CM, Mahomed N. An audit of radiation doses received by paediatric patients undergoing computed tomography investigations at academic hospitals in South Africa. S Afr J Radiol. 2020;24(1):a1823. https://doi.org/10.4102/sajr.v24i1.1823

CTDI_vol_, volume-based computed tomography dose index; DLP, dose length product; DP, Du Plessis et al.; VDM, Van der Merwe et al.

## Limitations

The incidence of minor blunt TBI was not evaluated in this study as only eligible patients, according to the PECARN CDR subsequently referred for CT imaging, were included in the study population. Further local studies, with larger study populations, are required to evaluate the burden of minor TBI on the healthcare system. Potential eligible patients might have been omitted from this study due to an incorrect triage diagnosis in the PEU patient register. This study is limited by the small sample size, the accuracy of the medical notes and storage thereof at hospital records.

## Conclusion

Clinicians need to balance the benefits of a CT scan and the potential risks associated with exposing young patients to ionising radiation. In this audit, none of the patients classified as a very low risk had CT findings of a TBI or a ciTBI (*p* < 0.01). Had the PECARN CDR been implemented, CT imaging could have safely been avoided with no negative impact for the patient.

For patients classified at intermediate risk, clinicians should engage parents and patients in their decision-making processes and consider the various clinical factors as set out by the PECARN CDR to ascertain whether observation or CT imaging is appropriate for the patient. Further prospective studies are needed to evaluate CT utilisation and understand the discrepancy in the expected and the actual association between intermediate and high-risk groups and CT findings of a TBI and ciTBI observed in this study.

Radiologists should always adhere to the ALARA principle and ensure that studies are justified. Diagnostic reference levels in our study were higher than local DRL standards. We recommend that institutions regularly audit their DRL levels, optimise paediatric imaging protocols and train staff in paediatric imaging to prevent unnecessary exposure to ionising radiation.
